# An examination of Australian newspaper coverage of the link between alcohol and cancer 2005 to 2013

**DOI:** 10.1186/s12889-017-4569-0

**Published:** 2017-07-24

**Authors:** Jaklin Eliott, Andrew John Forster, Joshua McDonough, Kathryn Bowd, Shona Crabb

**Affiliations:** The University of Adelaide, Adelaide, South Australia 5005 Australia

**Keywords:** Alcohol, Cancer, Australia, Newspaper, Public health, Advocacy

## Abstract

**Background:**

Alcohol is a Class-1 carcinogen but public awareness of the link between alcohol and cancer is low. The news media is a popular, readily-accessible source of health information and plays a key role in shaping public opinion and influencing policy-makers. Examination of how the link between alcohol and cancer is presented in Australian print media could inform public health advocacy efforts to raise awareness of this modifiable cancer risk factor.

**Method:**

This study provides a summative qualitative content analysis of 1502 articles that included information about a link between alcohol and cancer, as reported within Australian newspaper media (2005–2013). We use descriptive statistics to examine the prominence of reports, the nature and content of claims regarding the link between alcohol and cancer, and the source of information noted in each article.

**Results:**

Articles were distributed throughout newspapers, most appearing within the main (first) section. The link between alcohol and cancer tended not to appear early in articles, and rarely featured in headlines. 95% of articles included a claim that alcohol causes cancer, 5% that alcohol prevented or did not cause cancer, 1% included both. Generally, the amount of alcohol that would cause or prevent cancer was unspecified or open to subjective interpretation. Coverage increased over time, primarily within community/free papers. The claim that alcohol causes cancer often named a specific cancer, did not name a specific alcohol, was infrequently the focus of articles (typically subsumed within an article on general health issues), and cited various health-promoting (including advocacy) organisations as information sources. Articles that included the converse also tended not to focus on that point, often named a specific type of alcohol, and most cited research institutions or generic ‘research’ as sources. Half of all articles involved repetition of materials, and most confirmed that alcohol caused cancer.

**Conclusions:**

Information about a link between alcohol and cancer is available in the Australian newsprint media, but may be hidden within and thus overshadowed by other health-related stories. Strategic collaboration between health promoting organisations, and exploitation of ‘churnalism’ and journalists’ preferences for ready-made ‘copy’ may facilitate increased presence and accuracy of the alcohol-cancer message.

## Background

The news media is an important source of health information for many Australians, and plays a key role in shaping public opinion and influencing policy makers [1, 2]. Public health organisations have recognised the importance of harnessing the news media’s reach as part of a wider approach to distributing health information and advocating for changes in health policy [1, 3]. Past research has suggested that, compared with the expense and time restrictions of large-scale mass media campaigns (e.g., *Pink Ribbon Day*), influencing the media through direct media advocacy (e.g., through the use of media releases and developing relationships with journalists) is a low-cost approach that has the capacity to develop an ongoing discussion about health with the public [4, 5].

In recent years, raising awareness of cancer risk factors has become an important focus of public health advocacy [6]. Some of this has focused on increasing the coverage in the Australian news media of modifiable risk factors that, according to the World Health Organisation, are the cause of 30% of cancer cases: tobacco smoking, a lack of physical activity, low fruit and vegetable intake, having a high body mass index, and alcohol consumption [7].

Stories about cancer and its risk factors are frequently reported in the news media [8], as are stories about alcohol [9, 10]. However, news media typically frames the issues presented, emphasising certain points and omitting others, such that key information may be contested, obscured, unclearly or inaccurately presented, or simply absent [11]. In this context, this can be detrimental to public health messages regarding cancer and alcohol, particularly if such coverage is driven by alcohol companies and other stakeholders who benefit from the public consumption of alcohol, as occurred in the context of tobacco [12, 13]. Nonetheless, within Australia, the prevalence of newspaper articles promoting alcohol and alcohol consumption has increased, whilst those indicating disapproval of (harmful) alcohol use has decreased, suggesting a shift within the Australian public in the perception of alcohol use overall [10].

There is growing evidence that alcohol is linked with breast [14], colorectal [15], and prostate cancer [16]. Within Australia, estimates of annual incidence of alcohol-caused cancers vary from 2.8% [17] to 5.8% [18], with total numbers annually reported to range from 2182 to 6620 [17–19]. Despite this, a survey published in 2014 by the Foundation for Alcohol Research and Education found that less than one-third of Australians are aware of the link between alcohol and cancer [20]. This may be, in part, a result of the information to which Australians have been exposed in the news media. A growing academic literature has examined what Australians can read in the news media about cancer, including about risk factors [e.g., 5, 8]. However, to our knowledge, there is no such assessment of how the link between alcohol and cancer has been reported in the Australian (or any other) print news media, prompting the present study.

Historically, print newspapers are the medium of record and have been in a position to set public agendas [21]. Over time, there has been a decline in the circulation and economic viability of newspapers due in part to the rise of broadcast news and the growth of freely available news on the internet [22–25]. Nonetheless, newspapers have remained an influential part of the media landscape, often driving the bulletins of broadcast news and being appropriated as content for online discussion [26]. Furthermore, even if print newspapers ceased to be economically viable, the growth of subscription-based access to online newspaper content suggests that newspapers will remain relevant into the foreseeable future [25, 27].

There are several conventions of newspaper design and structure which are relevant to this analysis, providing valuable information on the prominence and content of articles not available in broadcast or online news media. As many readers scan a newspaper, rather than read it [28], editors structure newspaper content so as to increase the prominence of news that is deemed more important [29]. This is typically done by placing important information closer to the front page, and on odd-numbered (right-hand) pages (as this is where the gaze falls most readily). In addition, news articles focus on one topic, a ‘specific event, issue, person, group, or thing’ ([30], p. 42) that is considered newsworthy and potentially interesting to readers [28]. Bold and large headlines are an important means of attracting a reader’s attention and conveying the general topic, and are, in some cases, the only part of an article that is read [27].

In general, the longer an article, the less likely that it will be read in its entirety: For example, an American study [31] found that readers would only complete approximately 66% of a tabloid newspaper article 11 column inches (28 cm) in length (~385–440 words [32]), and approximately 56% of an broadsheet newspaper article of the same length. Mindful of limited space considerations and the potential for an article to be cut so that it fits on a page, journalists tailor their writing to be information-dense [28]. In practice this means that a general overview of the topic appears in the first paragraphs of an article, with other information building on the story included as the article progresses [33].

Finally, newspaper journalists produce news in a contested space between editors and their sources. At one end of the spectrum, editors prioritise the commercial imperatives of the newspaper and, as a result, sometimes engage journalists to focus on the unusual and sensational in health reports so as to pique reader interest and, ultimately, sell newspapers [2]. At the other end, sources seek to have their stories told in a way that is consistent with the narrative they wish to convey [29]. Journalists are reportedly careful to maintain their independence and not appear as a ‘mouthpiece’ for their sources [34–36], are concerned with the accuracy of their reports, and, in the case of sometimes complex health news, recognise the importance of including independent and respected experts to counterbalance potentially sensational aspects of a report [2]. Nonetheless, within Australia, as elsewhere, many media stories appear to consist of ‘recycled’ news often based upon materials produced by news agencies or public relations departments (including health advocacy organisations) [37, 38]. Whilst this may be (and often is) decried [38], it may be unrealistic to expect otherwise, given both declining staff numbers within journalism [38] and increasing restrictions of what organisational representatives are authorised to say, such that seeking further information would merely replicate the materials provided (e.g., media releases) [39].

It is well established that content analysis of health news reports can support public health advocacy by determining the pattern and framing of previous reports of a particular health issue [1, 3]. Content analyses can determine the frequency and prominence of information about a particular issue included in past news reports and the predominant news actors consulted by journalists [1, 3, 40]. Analysis of previous reports can also identify dominant frames, in particular, what and how problems are framed, as well as attribution of causality about a particular health issue as reported by journalists [1]. Where news information has been deficient or not met public health aims, public health advocates can use this knowledge to develop ways of introducing more effective messages into the news cycle [3], particularly, accurate, unambiguous, and straightforward messages that communicate health news from a public health perspective [2]. In the case of the link between alcohol and cancer, Cancer Council Australia stated in a recent position statement that alcohol use is a cause of cancer, and that *any* level of alcohol consumption increases the risk of developing an alcohol-related cancer ([18], p. 479).

In this paper, we present our analysis of how the link between alcohol and cancer was reported in the Australian newspaper media over a nine-year period from the 1^st^ January 2005 to the 31^st^ December 2013. Specifically, we sought to determine, within Australian newspapers: (i) the frequency and nature of reports of a causal link between alcohol and cancer, and whether articles focused on this link, (ii) the prominence of articles providing information about the link (within the newspaper), (iii) the prominence of information about the link within articles, and lastly, (iv) the frequency and nature of dominant sources of information cited within articles.

## Method

### Sample period

The decision to begin the sample period at the start of 2005 was based on the finding that the risk of cancer from alcohol was mentioned 37 times in the World Health Organisation’s 2004 Global Status Report of Alcohol [41], as compared with a brief, single sentence, mention in the earlier 1999 report [42]. This suggests that information regarding risk of cancer from alcohol, while available previously, may have become more integrated into the scientific literature from this time, actively promoted by public health advocates, and generally more accessible to the news media.

### Search

We used the term “alcohol AND cancer” to search 354 Australian newspaper titles included in the Dow Jones Factiva database between the 1st January, 2005 and 31^st^ December, 2013. The initial search identified 8177 articles. These were read by author AF, and those not featuring any link between alcohol and cancer were excluded (e.g., a story on Michael Jackson accused of molesting a boy with cancer, who allegedly had been plied with alcohol [43]). Those featuring some discussion of a link between alcohol and cancer were included in the dataset, and entered into an Excel database for coding and descriptive analyses.

### Measures

We applied a summative qualitative analysis [44] to assess the prominence and nature of the claims made, noting the following: date and type of newspaper mentioning the claim; location of article in newspapers, and claim in articles; and, content-specific features of articles: nature of the claim, the main topic (or focus, including whether this did or did not focus on alcohol, cancer, both, or neither), headline, word-count, descriptors of alcohol and cancer, and, information sources (see Table 1). An initial coding framework was established through coding every 10^th^ consecutive article, with the framework reviewed and refined by authors JE, KB, and SC, before coding the remainder. Data coding and checking, and specific analyses were carried out by AF, JE, and JM; any differences in coding or analysis were resolved through discussion, and, where necessary, review of the original cited article. All figures are rounded to the nearest digit. On download from Factiva, some information (e.g., formatting) was lost; all calculations are based upon available information.

**Table 1:** List of variables used to code 1502 articles presenting information regarding a link between alcohol and cancer

Claim	• Alcohol causes cancer; Alcohol does not cause cancer or prevents cancer
Mentioned in headline	• Alcohol and cancer; Alcohol only; Cancer only; Neither alcohol nor cancer
Cancer/alcohol focus in article	• On alcohol-cancer story or not (determined by review of headline, lead paragraph, percentage of story in article, and content)
Topic of article	• Focus of the article (determined by review of headline, lead paragraph, and content)
Page number (*n* = 1362)	• ≤ 15, > 15; Even or odd
Word-count	• Total word- count, median, and range of each article
Location in the article	• Where the link is reported: top, middle, bottom (Total word-count divided equally)
Location within the newspaper (*n* = 1348)	• Main section (not labelled) • Main section with feature headings if noted (e.g. BodyWork, Extra, Health & Fitness, Insight, Life, Opinion, Newsworld, Talking Point, World) • Other Sections (including supplements) not labelled • Supplements/lift-outs including names (e.g. Body & Soul, Feeling Great, Lifestyle, Magazine, Men’s Health, Taste, Opinion, Weekend)
Descriptive terms for (types of) alcohol	• Generic terms such as non-specific ‘alcohol’ or ‘drink’ • Named types of alcohol (e.g. beer, wine, vodka)
Descriptive terms for cancer	• Non-specific ‘cancer’; Named cancers (e.g., breast, colorectal, prostate) (nb: head & neck, oesophageal, larynx, mouth, throat, oral collated as ‘Head & neck; Bowel, colon, rectal, colorectal collated as ‘Bowel’)
Descriptive terms for alcohol amount	• The amount of alcohol associated with the development of cancer (e.g. use, consumption, binge drinking)
Source of information	• Dominant source of information mentioned or quoted (e.g. public health organisation, government agency, research journal or report, university, or charity exclusively or most frequently mentioned) (nb: data collated into categories based on the key function or focus of the organisations (e.g. (anti)cancer/alcohol advocacy, other health organisations, educational entities, health services (i.e. with interest in public health), generic, industry, or miscellaneous sources (i.e. other interests)
Date	• Date of publication

## Results

Over the nine-year period, 197 newspaper titles included at least one article mentioning a link between alcohol and cancer, with 1502 articles meeting inclusion criteria. These were most commonly printed in state daily (*n* = 558), followed by regional daily (*n* = 304), free community (*n* = 278), state Sunday (*n* = 229), national (*n* = 68) and, finally, rural non-daily (*n* = 65) newspapers. There was a general upward trend in the number of articles published annually, increasing from 119 in 2005 to 182 in 2013, with spikes in 2009 (*n* = 229) and 2011 (*n* = 271). Most of the increase occurred in free community, regional, and rural newspapers.

### Frequency and prominence of discussions regarding alcohol and cancer

Overall, 95% (*n* = 1425) of all articles included a claim that alcohol causes cancer (hereafter: *causative* or **A**➔**C** *articles*), 6% (*n* = 93) that alcohol prevents or does not cause cancer (hereafter: *non-causative* or **A ≠ C** articles), and 1% (*n* = *16*) included both claims (hereafter: *both non/causative* or *both* articles). Few articles (16%, *n* = 244) explicitly focused on a link between alcohol and cancer (hereafter called the *alcohol-cancer or A/C story*); most included this information in coverage of other, typically health-related topics. Of those articles focussing on the alcohol-cancer story, most (*n* = 228) were causative, with annual numbers ranging from 11 to 75, with an average of 13 (i.e., just over one per month); numbers rose from 11 in 2005 to peak at 75 in 2009, then declining to 11 in 2013. Over time there was an increase in the ratio of causative to non-causative articles (85:15 in 2005; 95:5 in 2013).

As shown in Table 2 Section a, approximately two-thirds of causative articles were located within the first 15 pages (of main and supplement sections) and just under half on odd-numbered pages; few featured on front pages (*n* = 6). Just over half of non-causative articles appeared within the first 15 pages of individual sections, and less than half on odd-numbered pages (see Table 2 Section b); none appeared on a front page.

**Table 2:** Location and repetition of stories, including and focusing on claims about a link between alcohol and cancer (by page number, and news section), and headlines

a. Article includes message that Alcohol causes Cancer [A➔C] (*N* = 1425)*									
Page detail	Focuses on A➔C (n = 204)^1^				Does not focus on A➔C (n = 1055)^2^			Total all (% all) (n = 1259)	
	Main section (*n* = 193)	Separate Lift-outs (*n* = 11)	Total (%)		Main section (*n* = 926)	Separate lift-outs (*n* = 129)	Total (%)		
pp. 1–15	131	9	140 (69%)		526	84	610 (58%)	750 (60%)	
pp. 16+	62	2	64 (31%)		400	45	445 (42%)	509 (40%)	
Odd pages	101	4	105 (51%)		475	35	510 (48%)	615 (49%)	
Even Pages	92	7	99 (49%)		451	94	545 (52%)	644 (51%)	
b. Article includes message that Alcohol prevents or does not cause Cancer [A ≠ C](*N* = 93)									
Page detail	Focuses on A ≠ C (n = 19^3^)				Does not focus on A ≠ C (n = 53)^4^			Total all (% all) (n = 72)	
	Main section (*n* = 19)	Separate lift-outs (*n* = 0)	Total (%)		Main section (*n* = 42)	Separate lift-outs (*n* = 11)	Total (%)		
pp. 1–15	9	0	9 (47%)		17	8	25 (47%)	34 (47%)	
pp. 16+	10	0	10 (53%)		25	3	28 (53%)	38 (53%)	
Odd pages	7	0	7 (37%)		23	2	25 (47%)	32 (44%)	
Even pages	12	0	12 63%)		19	9	28 (53%)	40 (56%)	
c. Topic mentioned in article headline									
Topic in headline	Article focuses on alcohol/cancer story (n = 242)^5^				Article does not focus on alcohol/cancer story (n = 1252)^6^				Total all (% all) (n = 1494)
	Article states A➔C (n = 224)	Article states A ≠ C (*n* = 16)	Article states Both (*n* = 2)	Total	Article states A➔C (*n* = 1177)	Article states A ≠ C (*n* = 61)	Article states Both (*n* = 14)	Total	
Cancer	25	2	0	27	386	7	1	394	421 (28%)
Alcohol	56	7	1	64	218	33	7	258	322 (21%)
Neither	52	1	1	54	555	18	6	579	633 (42%)
Both	91	6	0	97	18	3	0	21	118 (8%)
d. Number of articles by repetition of a topic,^7^ and sources featured in repeated articles (*N* = 739 articles)									
No. of repetitions of topics (total topics) (total n = 172 topics)									
Information source in article^8^	2–5 times (*n* = 141)		6–9 times (*n* = 140)		12–15 times (*n* = 91)		>15 times (*n* = 93)	Total	
Alcohol advocacy^9^	7		1		0		1	9	
Cancer advocacy^9^	49		7		5		1	62	145^11^ Topics
Other health promotors ^9^	24		3		0		1	28	
Education^9^	37		8		1		1	47	
Health services^9^	4		0		0		0	4	
Generic^10^	24		2		1		0	27	32^11^ Topics
Industry^10^	2		0		1		0	3	
Misc.^10^	2		0		0		0	2	

*See missing data per cell in Footnotes 1–6

^1^p# missing in 24 articles; ^2^p# missing in 141 articles; ^3^p# missing in 2 articles; ^4^p# missing in 19 articles; ^5^headlines missing in 2 articles; ^6^headlines missing in 6 articles; ^7^no topics were repeated 10, 11, 16–18, 20–22, 25–26, or more than 27 times; ^8^some articles had more than one source featuring equally; ^9^agencies with public health interests; ^10^other/industry/unknown interests; ^11^numbers exceed total repeated topics as some articles had more than one source featured equally

**Fig. 1:**
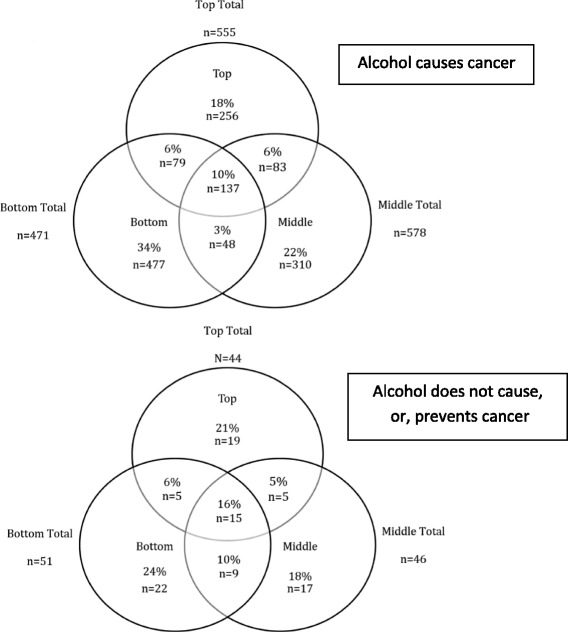
Location within articles (word-count divided into equal thirds) of claims regarding a link between alcohol and cancer

There were wide variations in word-count (22–4574) across the dataset with an average of 514, and a median of 382. The median word-count for causative articles was higher than for non-causative articles (381 and 324 respectively); a higher proportion of claims appeared earlier in non-causative articles than in causative articles (48% and 40% in the top-third of articles, respectively), though the overall number of latter far exceeded the former (see Fig. 1). (NB: 36 articles (3%) were excluded from the location-in-article analyses, due to formatting issues consequential on download from the newsprint database).

Most articles were situated in the main section with no specific heading (*n* = 930; 69% of total), followed by those in sections covering Feature/Extras (*n* = 142; 11%), Body/Health (*n* = 133; 10%), Opinion (*n* = 67; 4%), Life/style (*n* = 35; 2%), News/World (*n* = 34; 2%), and, Other (*n* = 7; <1%). Over time, there was a trend upwards in the proportion of articles published in the main section (2005: 55%; 2013: 77%), with others slightly decreasing or holding steady.

Articles with headlines including neither the word cancer nor the word alcohol were most common (*n* = 633), followed by those with the word cancer only (*n* = 421), the word alcohol only (*n* = 322), and those featuring both cancer and alcohol (*n* = 118). However, 82% of articles with both alcohol and cancer in the headline focussed on the alcohol-cancer story, compared with only 6% of those featuring just cancer, 9% of those with neither cancer nor alcohol, and 20% of those with just alcohol in their titles. Eight articles had no identified headline, only 2 focusing on the alcohol-cancer story (see Table 2 Section c ).

Almost half (*n* = 739) of all articles featured repeated topics. This was determined through comparison across coded variables for each article: topics were deemed repeated where articles appeared to be based on information from the same source, were published on the same day or successive days, focused on the same issue, and often had the same headline. 172 topics were the focus of two or more articles, and numbers of repetition of topics across articles varied from 2 to 27 (e.g., 57 topics were reported twice, and one topic was reported 27 times). Considering all articles featuring any repeated topic, relatively infrequent repetition (2–5 times) of 141 topics accounted for approximately 1 in 2 articles (total 415 articles); 19 topics were repeated 6–8 times (total 140 articles), 7 topics were repeated 12–15 times (total 91 articles), while topics repeated more than 15 times accounted for approximately 1 in 8 of these articles total (93 articles). Finally, the dominant sources of information within articles featuring repeated topics were entities likely to have an interest in promoting public health (see Table 2 Section d); for example, cancer advocacy organisations were named as sources of information in articles featuring 62 different topics. Most (691) articles with repeated topics included the claim that alcohol caused cancer, but only approximately one-quarter of these (162) focussed upon that claim (data not shown).

### The nature of specific links between alcohol and cancer

Of causative articles, more than 90% referred simply to ‘alcohol’ rather than specifying any type, where most **A ≠ C** articles (71%) did specify alcohol type, most often naming red wine, followed by beer, and strawberry daiquiri. By contrast, most causative articles specified particular cancers (66% naming particular cancers), whilst within the **A ≠ C** articles, the same proportion did not, rather using the generic term ‘cancer’ (see Table 3). The three most commonly discussed types of cancer (mentioned with other cancers and alone) were those proven to be causally linked to alcohol, and these were identical for both causative and **A ≠ C** articles; breast cancer was mentioned most often in causative, bowel cancer in **A ≠ C** articles, with head and neck following in both. Of the total, 456 articles referred simply to ‘alcohol’ and ‘cancer,’ with no further specification mentioned; all but 10 of these articles were causative.

**Table 3:** Frequency of ‘Alcohol’ (or alcohol subtypes), ‘cancer’ (or cancer subtypes), and descriptors of amount of alcohol mentioned in articles claiming a link between alcohol and cancer

CANCER and ALCOHOL (causes / does not cause Cancer) with subtype^1^ *n =* articles with exclusive mention of type / n = total articles mentioning type; % of totals within causative or non-causative articles for cancers only				
21/93 Alcohol >1 time	Causative articles (n = 1425)	Alcohol (1301/1406) Beer (9/48) Wine (30/78) Red (23/23) White/Riesling (7/7)	Mouthwash (29/29) Spirits (4/33) Whisky (5/5) Vodka (4/4) High-energy alcohol (2/2)	
	Non-causative articles (n = 93)	Alcohol (47/24) Wine (45/7) Red (38/37) White (5/0)	Beer (12/18) Strawberry Daiquiri (9/9) Whisky (1/6) Vodka (1/6)	
Cancers		Cancer only (472)		
	Causative articles	Breast (241/569) 40% Bowel (60/330) 23%^2^ Head & neck (89/309) 22%^3^	Liver (18/152) Prostate (12/116) Lung (4/116)	Skin or melanoma (2/89) Pancreatic (4/37) Cervix (2/52)
		Cancer only (52)		
	Non-causative articles	Bowel (3/13) 14%^2^ Breast (3/11) 12% Head & neck (0/6) 6%^3^	Prostate (11/14) Liver (0/2) Lung (1/5)	Skin or melanoma (0/1) Pancreatic (0/1) Cervix (0/3)
ALCOHOL DESCRIPTORS with examples^4^ *n* = total mentioned / *n = %* within causative or non-causative articles				
Alcohol link with cancer	Causative None in article: *n* = 531 (37%)	Non-specific (892/63%) (Consumption, alcohol intake, any amount, drink, use, consuming) Specific ≤2 drinks (28/2%) (small glass, 1.5 drinks daily, two drinks daily, one beer) Specific >2 drinks (57/4%) (more than 2 drinks, above 2 standard drinks, at least 3 drinks a day)	General low-moderate (42/3%) (little, modest, tipple) General heavy (240/17%) (too much, a bit of excess, more, exceeding, frequent, sustained) Problem labels (98/7%) (long heavy, excessive, alcoholism, dependency, chronic, abuse, binge, harmful) Other (20/1%) *(mouthwash, rising, habits)*	
	Non-causative None: n = 11 (12%)	Non-specific (55/\59%**)** (consumption, drink, preventative) Specific ≤2 drinks (12/13%) (two glasses of alcohol a day, one serving per week)	General low-moderate (8/9%**)** (moderate, light to moderate, light) General heavy (6/6%) (heavier, high, too much) Problem labels (1/<1%) (excessive)	

^1^Only subtypes mentioned more than once exclusively are included

^2^Bowel includes bowel, colon, rectal, colorectal

^3^Head & neck includes oral, mouth, oesophageal, pharynx, throat, tongue

^4^More than one descriptor possible in single article

As shown in Table 3, across the dataset, there was a great deal of variation in the language used to refer to the consumption of alcohol. A higher percentage of causative articles did not provide any terms to describe consumption (just noting that alcohol caused or was a risk factor for cancer) than did **A ≠ C** articles (37% and 12% respectively). Few descriptors of alcohol consumption were specific (e.g., more, or less, than a given amount), most constituting generic terms, with the majority open to subjective interpretation.

### Sources of information

The most common type of sources or authority figures within the dataset, comprising 38% of all articles, were those connected with cancer, either as advocates, agencies, or researchers in the field (see Table 4). The proportion of articles citing such authority figures that were focused on the alcohol-cancer story was markedly higher, at 57% of these articles. Together with those articles naming the equivalent entities for alcohol, such articles collectively comprised 43% of all articles, but 64% of those focussing on the alcohol-cancer story. A similar pattern held for articles featuring a specific educational or research agency (excluding any in the previous mentioned categories), featured in 16% and 23%, of all articles, and alcohol-cancer story-focused articles, respectively. By contrast, the proportion of those citing other health promoting agencies/researchers (though fewer) was higher within the total database, than in articles focused on the alcohol-cancer story (9% and 5% respectively), with identical figures evident for articles mentioning generic professionals/experts. One-quarter of all articles used generic terms, with about a third of these (9% of the total) using generic descriptors indicating some expertise or professional qualification; the remaining 16% did not specify the source beyond generic terms such as ‘report’ or ‘reporter.’ Only 5% of articles focussing on the alcohol-cancer story referred to generic sources, all being within the expert/professional category. Few articles (<2% overall), and none in the alcohol-cancer focused stories, cited industry as primary sources; one-third of these cited alcohol or consumer interest entities.

**Table 4:** Dominant authoritative information sources (by categories) mentioned across all articles (with examples), and in those focusing on the alcohol-cancer story

Type of Authority	Examples (most prevalent named first, with n provided)	ALL (*N* = 1502) *n* (%)	FOCUS (*n* = 244) *n* (%)
Alcohol/cancer advocacy (including relevant journals)	Cancer advocacy/agencies e.g. Cancer Council (*n* = 256, includes state and national councils), Cancer Australia, Journal of Clinical Oncology, National Breast Cancer Foundation, Queensland Cancer Fund, World Cancer Research Fund, US National Institute on Alcohol Abuse.	564 (38%)	140 (57%)
	Alcohol advocacy/agencies/research e.g. Salvation Army (*n* = 48), Alcohol Policy Coalition, Alcohol Education and Rehabilitation Foundation, Centre for Alcohol Policy Research.	87 (6%)	15 (6%)
	Both alcohol and cancer advocacy/agencies	652 (43%)	155 (64%)
Education (not including alcohol or cancer)	University of NSW (13), Oxford University, Queensland Institute of Medical Research, University College London, Journal of Science & Food Agriculture.	242 (16%)	55 (23%)
Other health promoting organisations	Australian Medical Association, British Health Department, Department of Veteran’s Affairs, Public Health Association of Australia, CSIRO, Australian Dental Association.	143 (9%)	13 (5%)
Health services/ treating orgs	Mayo Clinic, Royal Hobart Hospital, Flinders Medical Centre, Brigham and Women’s Hospital, Norwest Private Hospital, Gumbuya Mental Health Service, Red Cross Blood Services	49 (3%)	8 (3%)
Generic	Generic non-specific e.g. Reporter (*n* = 214), reporter, celebrity	236 (16%)	0 (−)
	Generic Professionals/Experts e.g. Dr. (*n* = 55), chief executive, Japanese researchers, Canadian study, GP, authority, anti-aging expert, researchers, nutritionist, study, dentist	137 (9%)	13 (5%)
	All generic sources	373 (25%)	13 (5%)
Industries	General (i.e. no-alcohol interest agencies) e.g. Goodguys (*n* = 12), Gym, Nathan Lion, Caci, Private business, Meat and Livestock Australia, Pfizer	23 (2%)	0 (−)
	Wine/alcohol/consumer interest agencies e.g. Australian Wine Research Institute (*n* = 10), Australian Consumer Organisation, International Winehealth, Absolut Cut, Gecko Winery	14 (<1%)	0(−)
	All industry sources	37 (2%)	0 (−)
Miscellaneous	Adelaide, Barcelona, subject, US entity	7 (>1%)	0 (−)

## Discussion

This is the first study of the link between alcohol and cancer as reported in the Australian print newspaper media. Over nine years, relatively few articles—an average of 167 per year (or three articles/week)—included some assertion about a link between alcohol and cancer, most providing information that alcohol causes cancer, with less than one per month asserting that alcohol prevents, or does not cause, cancer.

Regardless of whether the content of articles stated or rejected claims that alcohol caused cancer, there appeared to be little structural difference in the positioning, and length of articles in this dataset, or in the location of these claims within articles, suggesting that both messages were considered equally newsworthy (or not). Although articles with **A ≠ C** claims presented such claims earlier than those including causative claims, perhaps suggesting that this is deemed more newsworthy, there were fewer **A ≠ C** articles, and the length of most suggests that both claims are likely to be read by viewers. Few articles featured headlines drawing attention to the link between alcohol and cancer, but most stories, irrespective of message, were located in the main section of the newspaper. However, more of those focusing on the alcohol causes cancer message appeared earlier than those that did not. This differs from those of a content analysis of alcohol stories in Californian newspapers which revealed that stories promoting alcohol use were often located in ‘soft-news’ sections (e.g., in lifestyle, food, or wellbeing sections often within newspaper lift-outs) [45]. Claims about the health benefits or otherwise of alcohol may have appeal for Australian journalists seeking popular lifestyle stories about living a healthy life [46], but may also reflect normative expectations that individuals are or should be interested in taking action to maximise their health [47]—such that health could be deemed mainstream news. Paradoxically, this might also, in part, account for the finding that information on the alcohol-cancer link was rarely the focus of stories, but was most typically located within discussions of various health issues, or through including alcohol in a list of known cancer risks. A content analysis of 5327 articles about cancer in United States newspapers also reported that cancer risk factors (such as alcohol) and cancer prevention were rarely the main focus of news articles [48]. Instead, articles consistently focused on cancer treatment, which, the authors cautioned, offered drama and human interest, thus satisfying journalists’ focus on news values and the public’s news appetite, but framed cancer as something to react to, rather than prevent [48]. Similarly, researchers examining coverage of alcohol, health, and policy in 612 Australian television news stories observed that alcohol ‘problems’ were a common focus, but that only 10% of stories mentioned long-term health risks such as cancer, with little discussion of effective solutions [9]. These authors and others have noted a discrepancy between the news values of journalists and the goals of public health advocacy to improve health behaviours and change public health policy [2, 9, 48]. This discrepancy is certainly evident in those articles within the present study including a claim that alcohol prevents or does not cause cancer. Indefinite descriptors of the amount of alcohol that might cause cancer also implied that at least some use of alcohol was acceptable. As most individuals subjectively interpret drinking guidelines [49] and drink alcohol in accordance with personal attitudes and social mores (which may exceed levels recommended in Government guidelines), media representation of the link between alcohol and cancer may both reflect and reinforce the ongoing acceptance of alcohol in Australian society, and the perception that alcohol harms are more distal and not a problem for ‘responsible’ moderate drinkers [50]. To some extent, the variation in representations of the link across the dataset (i.e., in the descriptors of alcohol, cancers, and level of in/appropriate consumption) may contribute to the weakening or obfuscation of the unequivocal public health message that any and all consumption of alcohol increases cancer risk—regardless of the type of alcohol consumed [17]. There remains an ongoing need to effectively inform adults of the long-term cancer risks of low-level alcohol use, including cancer risk, such that this might potentially change drinking behaviours [51–53].

Somewhat reassuringly, however, the total annual coverage of reports about a link between alcohol and cancer increased over time, while the incidence of articles claiming that alcohol prevented or did not cause cancer decreased. This result supports findings by Azar et al. [10] that, within Australia, positive stories about alcohol consumption within the newsprint media stories have declined in frequency. These authors suggested this decrease could reflect a shift in the perceptions of Australians regarding the (harmful) use of alcohol. It is possible too that increased news media coverage of alcohol-related harm, including the long-term risk of cancer, may not only reflect, but contribute to the shift, as postulated in the case of tobacco-control [54].

Most articles that included a claim that alcohol causes cancer did not mention a specific alcohol, but, as has been found in past content analyses of news about cancer, most often focused on breast cancer [8]. This may have the unwitting effect of gendering public perceptions of alcohol-related cancer risk, such that women’s consumption of alcohol may be viewed as more problematic than that of men, despite evidence that rates of cancer [55] and alcohol [56] consumption are higher in men than women. Nonetheless, Pandeya et al. noted that rising alcohol consumption in young women could lead to increased numbers of cancers of the breast and other organs in future [17], indicating the importance of raising awareness of alcohol as one of the few modifiable risk factor for cancer, particularly for women with a known increased risk relative to the population as a whole (e.g. with familial history of breast cancer).

In general, non-causative articles were more likely (compared to causative articles) to specify an alcohol type, but less likely to specify particular cancers involved, and more likely not to name the source of featured information. Appearing annually from 2005 onward, red wine was the alcoholic product most often claimed to prevent cancer (e.g. *Score one for fickle red wine* [57]) echoing previous findings that red wine is often linked to health benefits in news reports [10]. One alcohol type, strawberry daiquiris, was featured in 9 different articles within one week (e.g. *Strawberry daiquiri, just what the doctor ordered* [58]). Such coverage could be examples of ‘churnalism,’ a common media practice [37–39] that saw materials apparently from a single source repeated across a wide variety of newspapers (e.g. state daily, state Sunday, and free community): in this dataset, almost half of all repeated topics featured more than two times.

Although the nature of the data means that it is not possible to determine the extent to which original material from a source was reproduced, we suggest, however, that churnalism can be harnessed to good effect by public health advocates, as the five most commonly-repeated stories featured messages that were endorsed by, and sourced from, public health advocates (e.g. *Reduce your risk, be “breast aware”*: Cancer Council SA [59]; *Oral cancer on the rise*: Australian Dental Association [60]; *Salvos call for alcohol warning*: Salvation Army [61]; *Alcohol blamed for more cancers*: Cancer Council Australia [62]. (nb. the release of the Cancer Council’s position statement on this likely explains the spike in numbers, as commentary both negative and positive followed dissemination of this information through the media); and *Mouthwash cancer*: Dental Journal of Australia [63]). All four (and indeed the strawberry daiquiri story) exhibited characteristics that identified them as ‘newsworthy stories’ (referring to powerful organisations, with an element of surprise, bad or good news involving large numbers of people, and/or follow-ups ([64], p. 279), but this practice provides some leverage for public health advocates seeking to increase newspaper coverage of public health information. For example, coverage of the most repeated story—that mouthwash was carcinogenic—featured disagreement amongst health professionals, a well-selling product threatened with removal from circulation, commercial interests, purported conflicts of interest, and ‘scary’ statistics. Whilst each factor individually might attract media attention, the juxtaposition of all, and particularly the professional debate over the significance or accuracy of reported findings most probably explains the frequency and longevity of the story (Jan-Dec 2009)—and accounts for the overall spike in alcohol-cancer coverage in 2009. As also reported by an Australian study which demonstrated that targeted media engagement by public health advocates prompted increased coverage of anti-tobacco news stories [29], this suggests that providing comment on current news stories with public health relevance within the media, and perhaps especially those that contradict preferred messages, can garner extensive coverage with relatively little cost or effort.

It is plausible that the frequent citation, within the database, of information sourced from advocacy organisations, health organisations, or research/education bodies likely to have a positive interest in public health indicates both journalists’ efforts to seek the opinion of relevant experts, and the said experts’ efforts to pro-actively disseminate information to promote public health [65, 66]. However, whilst many different sources were cited overall, cancer advocacy organisations were the dominant source in articles including a claim that alcohol causes cancer, with relatively little contribution from other domains, including from their alcohol counterparts. This has implications for public health advocacy. For example, in the public health movement against tobacco, the diversity of voices and frequent mention of various professional groups (e.g., the Australian Medical Association), social movement activist groups (e.g., the Non-Smokers Movement of Australia), and non-government public health organisations (e.g., the Heart Foundation) helped build and solidify negative public perceptions of tobacco, which ultimately, fostered an agenda for changing tobacco policy [57]. The effective partnership of the Salvation Army, who professed support for the Cancer Council’s call for alcohol warning labels (e.g. [61]) point to the potential for public health advocates to promote (more) collaborative news media campaigns with diverse others, thus increasing overall media coverage.

## Limitations and future research

Given the limitations of the Dow Jones Factiva database, we could not ascertain the exact placement of an article on the newspaper page, how much space it occupied, the inclusion of graphics, and any other stories and advertisements present on the page (especially major events). Examination of these factors could provide a further indication of the prominence of the article within the paper, and the message regarding the link between alcohol and cancer within the article [67]. Future analyses might also consider comparison between how alcohol is reported before and after the World Health Organisation’s 2004 Global Status Report of Alcohol [40], and with regard to other modifiable risk factors for cancer (e.g. tobacco smoking, diet). Finally, an in-depth qualitative analysis of the framing of, and specific use of language within, these stories may be a useful avenue for future research, providing further insight into why many Australians remain unaware of that alcohol causes cancer.

## Conclusion

Our results suggested that the claim that alcohol causes cancer has been regularly included across the Australian newspaper media, but this message may be obscured within or overshadowed by other (potentially competing) health information. The impact of the message that *any* consumption of *any* alcohol (regardless of amount or type) will increase risk of cancer is also potentially obscured by language that suggests or implies that there is a safe level of alcohol consumption, or that alcohol does not cause, and can prevent cancer. The increasing use of ‘churnalism’ by journalists may provide opportunity for future public health advocacy efforts to reduce cancer risk at a population level by offering ‘copy’ that meets media values that include not just to inform, but to entertain [64, 68]. Further strategies to disseminate information about the link between alcohol and cancer in a way that increases its newsworthiness, and therefore prominence could perhaps be achieved through strategic collaboration with other organisations addressing population risk-reduction through life-style change, with an aim to ensure consistency of message with diversity of voices. The provision of public health information for inclusion in news media will also need to accurately represent alcohol-related cancer risk, critically without alienating an audience wherein consumption of alcohol is normative. As noted elsewhere [52, 53], this will require an in-depth understanding of how such messages are responded to and/or acted upon by news recipients.
